# *DMRTC2*, *PAX7*, *BRACHYURY*/*T* and *TERT* Are Implicated in Male Germ Cell Development Following Curative Hormone Treatment for Cryptorchidism-Induced Infertility

**DOI:** 10.3390/genes8100267

**Published:** 2017-10-11

**Authors:** Katharina Gegenschatz-Schmid, Gilvydas Verkauskas, Philippe Demougin, Vytautas Bilius, Darius Dasevicius, Michael B. Stadler, Faruk Hadziselimovic

**Affiliations:** 1 Cryptorchidism Research Institute, Kindermedizinisches Zentrum Liestal, 4410 Liestal, Switzerland; liestal@kindermedizin-zentrum.ch; 2 Children’s Surgery Centre, Faculty of Medicine, Vilnius of University, 01513 Vilnius, Lithuania; gilvydas.verkauskas@vuvl.lt (G.V.); vytautas.bilius@vuvl.lt (V.B.); 3 Biozentrum, Life Sciences Training Facility, University of Basel, 4001 Basel, Switzerland; philippe.demougin@unibas.ch; 4 Institute for Pathology, National Centre of Pathology, Affiliate of Vilnius University Hospital Santariskiu Klinikos, 08406 Vilnius, Lithuania; darius.dasevicius@vpc.lt; 5 Friedrich Miescher Institute for Biomedical Research, 4058 Basel, Switzerland; michael.stadler@fmi.ch; 6 Swiss Institute of Bioinformatics, Basel, Switzerland

**Keywords:** gonocytes, Ad spermatogonia, RNA-sequencing, testosterone, LH, GnRHa-treatment, infertility, cryptorchidism

## Abstract

Defective mini-puberty results in insufficient testosterone secretion that impairs the differentiation of gonocytes into dark-type (Ad) spermatogonia. The differentiation of gonocytes into Ad spermatogonia can be induced by administration of the gonadotropin-releasing hormone agonist, GnRHa (Buserelin, INN)). Nothing is known about the mechanism that underlies successful GnRHa treatment in the germ cells. Using RNA-sequencing of testicular biopsies, we recently examined RNA profiles of testes with and without GnRHa treatment. Here, we focused on the expression patterns of known gene markers for gonocytes and spermatogonia, and found that *DMRTC2*, *PAX7*, BRACHYURY/*T*, and *TERT* were associated with defective mini-puberty and were responsive to GnRHa. These results indicate novel testosterone-dependent genes and provide valuable insight into the transcriptional response to both defective mini-puberty and curative GnRHa treatment, which prevents infertility in man with one or both undescended (cryptorchid) testes.

## 1. Introduction

**Figure 1:**
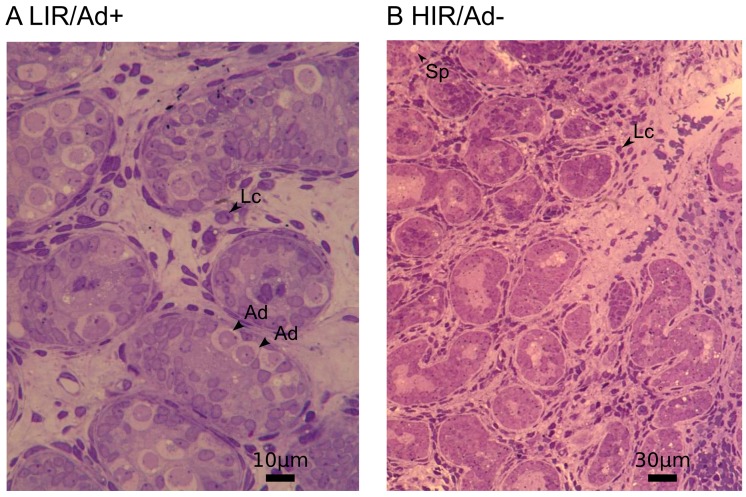
Semi-thin sections of prepubertal cryptorchid testes. (**A**) Low infertility risk (LIR/Ad+) testes and (**B**) high infertility risk (HIR/Ad−) testes. Atrophic Leydig cells (LC) and a severe reduction of germ cells is a typical picture in cryptorchid boys with defective mini-puberty (Ad− group). Dark type (Ad) spermatogonia, juvenile LC and germ cells (Sp) are indicated with arrow heads.

**Figure 2:**
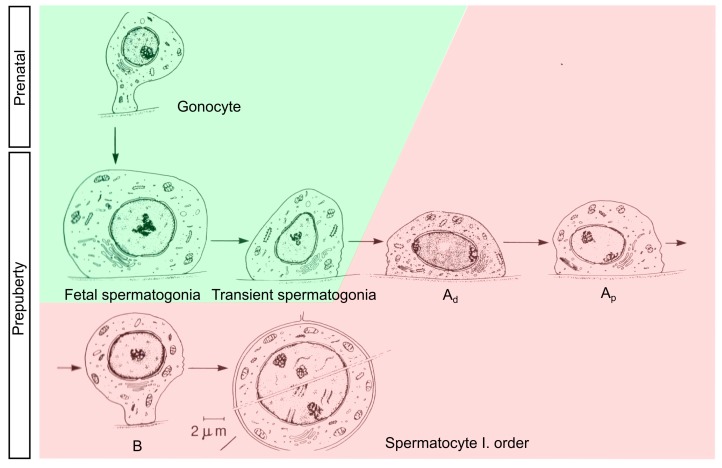
Male germ cell development. Differentiation of gonocytes into Ad spermatogonia is highlighted as color change from green to red (figure adapted from Hadziselimovic and Herzog [26]).

During mini-puberty, which occurs between 30 and 90 days of postnatal life in male infants, the substantial increase in gonadotropin releasing hormone (GnRH) secretion induces gonadotropin and testosterone production [1,2,3]. As a consequence, transformation of gonocytes into adult dark (Ad) spermatogonia takes place. Ad spermatogonia have a characteristic nuclear feature that distinguishes them from the other germ cells (e.g., fetal, transient, and pale-type (Ap) spermatogonia) (Figure 1 and Figure 2). Ad spermatogonia appear at three months of age and remain present in the testes for the rest of an individual’s life. Therefore, the transformation of gonocytes into Ad spermatogonia, either directly or through intermediate stages, is not simply another step in a succession of developmental stages, but a major transformation. It represents the switch from a fetal reservoir of stem cells (gonocytes) to an adult reservoir of stem cells (Ad spermatogonia), from which all future germ cells are generated [4,5,6]. Insufficient testosterone levels fail to direct gonocytes into the differentiation process in boys with defective mini-puberty, resulting in both abrogated Ad spermatogonia development and infertility [7,8,9].

Cryptorchid boys, of a median age of eight years, who were treated with a gonadotropin-releasing hormone agonist, showed post-puberty improved sperm concentrations, when compared to an untreated control group [10]. The treatment resulted in increased luteinizing hormone (LH) levels and regeneration of atrophic juvenile Leydig cells, and increased numbers of germ and Leydig cells [11,12]. Worth noting, long term follow-up in a high infertility risk group of cryptorchid boys, treated before the age of six, showed normal sperm concentrations in 86% of cases [13]. This result strongly contrasts with those of a ‘surgery only’ group, in which not a single patient had a normal semen analysis and 20% suffered from azoospermia [13].

Though expression patterns may differ, development in both humans and mice appear to involve a similar set of genes, which can also be used as markers to distinguish gonocytes and spermatogonia (reviewed in [14,15]). Because the transition process is similar between both species, animal models are commonly used, since the testicular tissues necessary to study this process in humans are difficult to obtain.

ALPP/PLAP, EPS8, KIT/c-KIT, NANOG, POU5F1 and TFAP2C/AP2γ are gonocyte markers, while *ALPP*, *NANOG*, *POU5F1*, and *TFAP2C* encode transcription factors known to be important for pluripotency. The differentiation of gonocytes into spermatogonia is associated with upregulation of certain genes, including *MAGEA4*, *DDX4* and *TSPY1* [16,17], while *POU5F1*, *ALPP*, *TFAP2C*, *NANOG* and *KIT* are downregulated [17,18,19,20,21]. Markers for self-renewal (ETV5, FOXO1, GFRA1, ID4, RET, SALL4, UTF1, CHD1L, and TAF4B) or differentiation (DMRT1, ZBTB16/PLZF, FGF9, FGFR3, NANOS2, NANOS3, DAZ1, DAZL, SOHLH1, SOHLH2, NEUROG3, and PHF13/SPOC1) can be used to identify undifferentiated spermatogonia. However, the contribution of these proteins to the testosterone-dependent transition as well as their mechanisms of action remain unclear.

In this study, we investigated the molecular events underlying human male germ cell development, focusing on the testosterone-dependent transition from gonocytes to Ad spermatogonia as well as the molecular impact of early GnRHa (Buserelin INN) treatment. Utilizing testicular gene expression profiles from testes with insufficient testosterone secretion, before and after GnRHa administration, and testes with completed mini-puberty, we identified the *DMRTC2*, *PAX7*, *BRACHYURY*/*T*, and *TERT* genes to be associated with defective mini-puberty and responsive to GnRHa.

## 2. Materials and Methods

### 2.1. Study Population and Biopsy Sample Collection

We selected 15 patients with isolated cryptorchidism, based on histological results, and divided them into 2 groups. Seven belonged to the Ad− (lacking Ad spermatogonia) and 8 to the Ad+ (presenting Ad spermatogonia) group. Data from Ad− bilateral cryptorchid boys treated with GnRHa (Buserelin) following the first orchidopexy (surgery) (4 patients) were retrieved from an ongoing randomized study. Initial biopsies revealed no Ad spermatogonia, indicating defective mini-puberty (Ad− group). The second testis was managed by orchidopexy and biopsied 6 months after the initial surgery. Thus, results from 19 biopsies were compared. Patients were age and ethnicity matched. RNA sequencing data from manually selected germ cell marker genes from our two previous studies [22,23] were analyzed.

A cryptorchid testis is defined as a testis localized outside of the scrotum and incapable of being brought into a stable scrotal position. All undescended testes in this study were located in the inguinal region. Testicular biopsies were taken at the time of orchidopexy. This sample was then subdivided, with one fragment fixed in glutaraldehyde for histological processing, while the other one was immediately immersed in RNAlater (ThermoFisher Scientific, Waltham, MA, USA) and stored at –25 °C until further processing (for RNA extraction and RNA- sequencing).

### 2.2. Histological Analyses

Biopsies were fixed in 3% glutaraldehyde in phosphate-buffered saline (PBS, pH 7.4) and then embedded in Epon resin. Semi-thin sections (1 μm) were cut using a Reichert Om-U3 ultramicrotome (Reichert AG, Vienna, Austria). Sections were mounted on glass slides, stained with 1% toluidine blue, and examined under a Zeiss Axioskop light microscope (Carl Zeiss Microscopy Gmbh, Jena, Germany) with an integrated photo-camera. Biopsies were histologically examined by two of the authors (F.H. and D.D.), each with expertise in the interpretation of semi-thin sections of prepubertal testes.

During histological analyses, at least 100 tubular cross sections per biopsy were evaluated, with regard to their number and absence of Ad spermatogonia. In the prepubertal testes, Ad spermatogonia were identified according to the criteria first published by Seguchi and Hadziselimovic [24]. This type of germ cell has a typical halo in the nucleus, termed the rarefication zone, and cytoplasm with a darker aspect in comparison to Ap or fetal spermatogonia.

### 2.3. RNA Preparation, Sequencing, Data Analyses, and RNA Expression Levels

The workflow from RNA isolation, through to purification, library preparation, sequencing, data analyses, and expression level analyses, has been described previously [22,23].

### 2.4. Data and Differential Gene Expression Analyses

Determination of differentially expressed genes, statistical analyses and model design were described previously [22,23]. Only genes with at least one read per million, in at least two samples, were included. p values and fold-changes were calculated for the treatment factor and differentially expressed genes were defined as those displaying a false discovery rate (FDR) of less than 0.05 and an absolute change in expression of at least two-fold. Raw data files are available at the Database of Genotypes and Phenotypes (dbGaP) with the accession number phs001275.v1.p1.

### 2.5. Protein Interaction Network

Two gonocyte marker genes, 19 spermatogonial marker genes and 8 putative spermatogonia genes, all of which are differentially expressed between the two groups (Ad− and Ad+), were used as input to obtain the protein–protein interaction network using STRING version 10.0 [25].

### 2.6. Ethics Statement 

Investigations were carried out in accordance with the Declaration of Helsinki of 1975, revised in 2008. All aspects of this study were approved by the Institutional Review Board and the Independent Ethics Committee of Vilnius University. Approval was also provided for research involving the use of material (data records or biopsy specimens) that had been collected for non-research purposes (Vilnius Regional Biomedical Research Ethics Committee, No. 158200-580-PPI-17, 11 June 2013).

## 3. Results

Here, we focused on selected marker genes for gonocytes and Ad spermatogonia (Table 1). 

Gonocytes are defined as small cells originating from the primordial germ cells and localized predominately in the center of the tubule and small typical mitochondria. They give rise to the fetal spermatogonia, which are the largest germ cells in prepubertal testis (Figure 2). This type of germ cells represents a population of so called undifferentiated spermatogonia. In the group of undifferentiated spermatogonia we included also all transient forms of the germ cells, which evolve into A spermatogonia [24].

### 3.1. Decreased Expression of Gonocyte and Spermatogonial Marker Genes in Testes with Altered Mini-Puberty

Of the six gonocyte markers selected, *POU5F1* and *TFAP2C* showed lower expression levels in Ad− testes with testosterone deficiency (Table 1), and 19 of 34 spermatogonial marker genes showed reduced expression in Ad− testes with insufficient testosterone levels. This group includes genes involved in spermatogonial stem cell (SSC) self-renewal (*ETV5*, *ID4*, *PAX7*, *RET*, *SALL4, BRACHYURY*/*T*, *TERT)* as well as mitotic-to-meiotic germ cell transition and differentiation (*DAZ1*, *DAZL*, *DDX4*, *FGF9*, *FGFR3*, *NANOS2*, *NANOS3*, *SOHLH1*, *SOHLH2*, *UCHL1*) (Table 1). These results confirm and extend previous GeneChips observations related to *ID4*, *DAZ1*, *DAZL*, *DDX4*, *FGF9*, *FGFR3*, in testes exposed to defective mini-puberty [27,28] and emphasize their importance in testosterone-dependent development into Ad spermatogonia. Additionally, the marker gene with unknown function (*MAGEA4*) was less expressed. Interestingly, 19 marker genes showed no difference in expression (*ADGRA3*, *ALPP*, *BCL6B*, *CDH1*, *CHD1L*, *DMRT1*, *EPS8*, *FOXO1*, *GFRA1*, *KIT*, *NANOG*, *NEUROG3*, *PHF13*, *POU2F2*, *TAF4B*, *THY1*, *TSPAN8*, *UTF1,* and *ZBTB16*). There were no increased RNA levels observed for any of the gonocyte and spermatogonial markers in testes without Ad spermatogonia (Table 1).

### 3.2. Gonocyte and Spermatogonial Marker Genes Respond to GnRHa Treatment

Out of the six gonocyte marker genes tested, *EPS8* and *KIT* showed decreased RNA expression after GnRHa treatment (Table 1). Neither gene was differentially expressed between testes, with or without Ad spermatogonia. *ALPP*, *NANOG*, *POU5F1*, and *TFAP2C* expression levels were similar between the treated and untreated testes, and GnRHa treatment did not lead to upregulation of any gonocyte markers.

**Figure 3:**
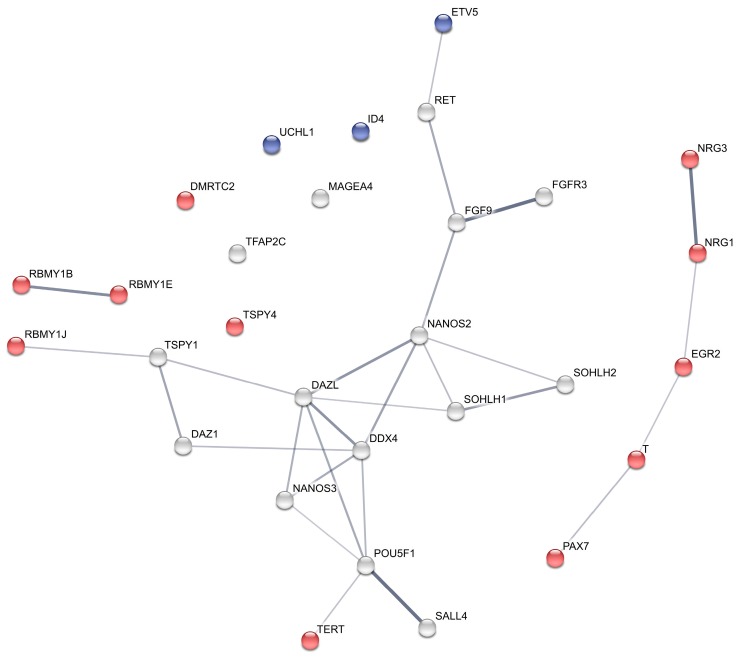
Protein interaction network of differentially expressed gonocyte and spermatogonial marker genes in Ad−/Ad+ and after GnRHa treatment. Protein coding genes which up- or downregulated after GnRHa treatment are represented by red and blue nodes, respectively. STRING was used to predict the protein interaction network [25] with a confidence cut-off of 0.4. Line-weight represents the strength of data support between the predicted interactions.

Downregulation was observed in nine of 34 spermatogonial marker genes (*ADGRA3*, *CHD1L*, *DMRT1*, *ETV5*, *FOXO1*, *ID4*, *TAF4B*, *THY1*, *UCHL1*), and the expression levels of *ETV5*, *ID4* and *UCHL1* were lower than in testes with Ad spermatogonia, indicating that GnRHa treatment further decreased the expression of these genes (Table 1 and blue nodes in Figure 3). The expression of 20 spermatogonial markers showed no significant response to GnRHa treatment (Table 1). Five spermatogonial genes (*PAX7*, *POU2F2*, *BRACHYURY*/*T*, *TERT*, *TSPAN8*) responded with an increase in RNA expression. *TSPAN8* was not differentially expressed between Ad− and Ad+ testes. *PAX7*, *BRACHYURY*/*T*, and *TERT* were both less expressed in Ad− testes and upregulated following GnRHa treatment (Table 1 and red nodes in Figure 3). These genes showed the strongest treatment effect.

Only a few of the marker genes downregulated in Ad– testes were then upregulated after GnRHa treatment. Therefore, we searched for additional genes involved in testosterone-dependent gonocyte-to-Ad spermatogonia transition (Supplementary Tables S1 and S2). We identified eight additional candidates matching these criteria: *DMRTC2*, *EGR2*, *NRG1*, *NRG3*, *RBMY1B*, *RBMY1E*, *RBMY1J*, and *TSPY4* (Table 1). A positive effect of GnRHa on *EGR2*, *NRG1*, *POU2F2*, *RBMY1B*, *RBMY1E* and *RBMY1J* gene expression has been previously reported [22,23].

We next interpreted the 29 markers (including putative markers, and all of which are differentially expressed between Ad− and Ad+) in the context of physical protein–protein interactions and functional interactions, by integrating our data with information available in the literature (STRING interaction network; http://string-db.org [25]). Markers responding positively to GnRHa (red nodes in Figure 3) are mostly not connected to the non-responsive (grey nodes) key germ cell markers such as FGF9, NANOS2, SOHLH1 and SOHLH2, suggesting that, at the protein level, GnRHa stimulates and activates alternative pathways in germ cells. This is consistent with our previous observations of alternate GnRHa responsive genes in the hypothalamus-pituitary-gonadal (HPG) axis [23]. Especially the markers, PAX7, BRACHYURY/T, EGR2, NRG1 and NRG3, seem to represent an alternative pathway, activated by GnRHa and involved in gonocyte-to-Ad spermatogonia transition. It should be noted that although not visualized by STRING, BRACHYURY/T expression was reported to be partially influenced by POU5F1 [29], and to be a downstream effector of GDNF/ETV5 signaling to promote self-renewal [30].

## 4. Discussion

### 4.1. Luteinizing Hormone and Testosterone Deprivation Decreases Gonocyte and Spermatogonial Marker Gene Expression

*POU5F1* encodes a transcription factor that plays a key role in both embryonic development and stem cell pluripotency. In human fetal gonads, *POU5F1* regulation differs in male and female germ cells. While POU5F1 expression is gradually downregulated during gonocyte differentiation, in human males, in females it is downregulated much earlier in fetal life, when the oocytes enter the first meiotic prophase [31]. TFAP2C/AP2γ expression is also gradually reduced during gonocyte differentiation [32]. The reduced levels of *POU5F1* and *TFAP2C* RNA, observed in Ad− testes compared to Ad+ testes, lead to the assumption that they play an important role in LH and in the testosterone-dependent gonocyte-to-Ad spermatogonia transition.

*ETV5* encodes a glial cell-derived neurotrophic factor (GDNF)-inducible transcription factor, regulating several genes known to be important for stem cell self-renewal, including *BCL6B*, *LHX1*, *CXCR4*, *BRACHYURY*/*T*, and *RET* [30,33]. The fact that *ETV5*-null mice are infertile demonstrates the importance of ETV5 for spermatogenesis [34]. Moreover, Wu and coworkers showed that transplantation of SSCs in vivo following *Brachyury*/*T* silencing significantly reduces the number of donor cell-derived colonies formed in recipient mouse testes, suggesting that BRACHYURY/T functions as a part of GDNF/ETV5 signaling to promote self-renewal [30]. BRACHYURY/T is a classical mesodermal marker, which is regulated by WNT and BMP signaling and expressed in early mouse and human primordial germ cells [35,36]. Ad− testes showed reduced expression levels for GDNF signaling factors—*ETV5*, *CXCR4*, *BRACHYURY*/*T*, and *RET*—which indicates disturbed GDNF-dependent self-renewal in the germ cells.

The pluripotency transcription factor, SALL4, was found to be localized to primordial germ cells and most gonocytes in the prenatal and early postnatal testes, as well as in undifferentiated spermatogonia of marmoset, human and mouse pubertal and adult testes [37,38]. SALL4 regulates the expression of genes required for either self-renewal or differentiation, along with ZBTB16 and DMRT1 [39].

NANOS2 and NANOS3 are RNA binding proteins from the NANOS family. In mice, a *Nanos3* deficiency results in the loss of primordial germ cells during migration and leads to sterility in male and female mice [40]. In contrast, a *Nanos2* loss results in decreased germ cell numbers and causes infertility in only male mice [40]. Murine NANOS2 activates a male-specific genetic program in female germ cells by inhibiting meiosis, which suppresses the female pathway and induces male-type differentiation [41]. Furthermore, *Nanos2* expression is directly dependent on retinoic acid (RA) for its downregulation and fibroblast growth factor 9 (FGF9) for its upregulation [42]. While *Nanos2* expression is restricted to prenatal germ cells and a small number of spermatogonia in adult mice [40,43], NANOS2 was reported to be more widely expressed in adult humans, including in spermatocytes and round spermatids [44]. Therefore, Kusz and colleagues suggested that NANOS2 is not a suitable spermatogonial marker in adult men, although our results indicate that NANOS2 could be a potential marker for spermatogonia in young boys. SOHLH1 and SOHLH2 together with DMRT1 and DMRT6/DMRTB1 ensure that meiosis starts only when spermatogonia have reached the appropriate differentiation step [45,46]. In summary, *Nanos2*, *Sohlh1* and *Sohlh2* all play substantial roles in male germline development in mice, and our results strongly support the notion that they fulfill the same role during mini-puberty in humans.

### 4.2. Genes with Augmented Levels after GnRH Treatment

Testosterone treatment of mouse satellite cells was shown to increase *Pax7* expression [47], supporting the present observation of decreased *PAX7* expression in Ad− testes with low testosterone levels as well as increased *PAX7* expression in the testes of boys with increased testosterone levels, following GnRHa treatment.

Lim and colleagues observed a predominant expression of the transcription factor POU2F2 in Ad spermatogonia [48]. Although it has yet not been confirmed by other studies in humans whether POU2F2 is indeed a specific marker for Ad spermatogonia, our RNA expression results after GnRHa treatment do support this finding.

High telomerase expression levels were found to be a hallmark of undifferentiated spermatogonia using telomerase reverse transcriptase (*TERT*) reporter mice [49]. It was also shown that telomere dysfunction caused undifferentiated spermatogonia depletion, which disrupted male germ cell development. While high telomerase expression has yet not been demonstrated as a hallmark for human spermatogonia, our differential expression data strongly support this possibility. Atrophic testes had lower *TERT* expression levels relative to normal testes, leading to the suggestion that telomerase plays a role in maintaining germ cell proliferation [50]. Furthermore, the *TERT* mRNA expression level was shown to be effective in both classifying spermatogenesis disorders in patients, and in predicting successful sperm recovery in azoospermia patients [51,52]. Androgens were reported to enhance *TERT* expression in human primary hematopoietic cells [53]. Calado and colleagues also demonstrated that the aromatase, CYP19, known to convert testosterone into estradiol, is necessary for the testosterone-dependent increase in *TERT* expression. This supports both, our finding of reduced *TERT* expression in lower testosterone Ad- testes, but also the observed increased *TERT* expression in Ad– testes with increased testosterone levels after treatment. Additionally, GnRHa treated testes showed significantly increased *CYP19A1* gene expression levels (absolute fold change logFC^GnRHa^ + 2.51) [23].

Neither testosterone, nor LH dependent gene expression of *BRACHYURY*/*T*, has yet been reported. However, *BRACHYURY*/*T* was shown to bind to the promotor of the androgen receptor (AR) and regulate AR expression in prostate cancer cells [54]. Increased *BRACHYURY*/*T* levels after GnRHa treatment point towards its role in Ad spermatogonia formation. This is a new observation which indicates this gene to be an important marker of Ad spermatogonia.

EGR2 and EGR3 are transcription factors used as spermatogonial markers, and EGR3 immunoreaction was reported in A single or paired germ cells in mice [55]. Furthermore, in mice *EGR3* expression is stimulated by RA and downregulated by KIT Ligand (KITLG) [56].

The neuregulins, NRG1 and NRG3, are essential for the proliferation of spermatogonia and the initiation of meiosis [57]. NRG1 and KITLG were also reported to activate alternative pathways downstream of RA signaling in the germline, known to be essential for spermatogonial differentiation [58]. Notably, after GnRHa treatment *NRG1* was upregulated, while *KITLG* was downregulated (logFC^GnRHa^ − 1.10).

While mouse *RBMY* is expressed only in spermatogonia and early spermatocytes [59] and its mRNA is not detected in meiotic or post-meiotic cells [60], human RBMY is expressed in spermatogonia, spermatocytes, and round spermatids [61,62]. Therefore, it is notable that testes lacking Ad spermatogonia showed significantly reduced RBMY RNA levels that increased strongly after GnRHa treatment.

We also found a treatment related increase in *DMRTC2/DMRT7* and *TSPY4* gene expression. *DMRT7* mutant mice show meiotic arrest at the pachytene stage [63,64], and DMRT7 protein is present in germ cells, localized to the male XY body during meiosis, and essential for male but not female fertility in mice [64]. While murine DMRT7 was predominantly expressed in mid-to late-pachytene spermatocytes and not detected in other germ cells including spermatogonia [64], our results point to a role of DMRTC2/DMRT7 in the early stage of human male germ cell development. Although the function of *TSPY4* is unknown, from sequence similarity it is assumed to be involved in sperm differentiation and proliferation (UniProtKB/Swiss-Prot, TSPY4_HUMAN, P0CV99). Interestingly, TSPY was reported to bind to androgen receptor and AR variants, and thereby increase the transactivation of the AR/AR-V7 target genes [65]. Whether testosterone-dependent TSPY4 does also bind AR is unknown.

### 4.3. Combining Classical Physiological Information and Cutting-Edge Genomics Data into a Complete Picture

Here, we report that testes with defective mini-puberty, with lower testosterone levels, and lack of Ad spermatogonia had significant lower RNA levels for selected gonocyte and spermatogonial marker genes (21 genes) relative to testes with Ad spermatogonia. We suggest that these differentially expressed genes reflect molecular functions involved in the gonocyte-to-Ad spermatogonia transition in humans during mini-puberty. Furthermore, we propose that higher expression levels of these 21 genes in testes presenting Ad spermatogonia are the result of testosterone-dependent expression, since the lack of testosterone increase during mini-puberty causes developmental arrest. The finding of four gonocyte markers and 15 spermatogonial marker genes that are not differentially expressed argues against a dilution effect and supports the importance of these findings.

*PAX7*, *BRACHYURY*/*T*, and *TERT* responded positively to GnRHa treatment, and were markers with reduced expression in Ad− testes. We suggest that the genes *DMRTC2*, *EGR2*, *NRG1*, *NRG3*, *RBMY1B*, *RBMY1E*, *RBMY1J* and *TSPY4* represent potential new markers for spermatogonia in infant testes, and that they may have key functions in the gonocyte-to-Ad spermatogonia transition. Additionally, it seems likely that they are testosterone-responsive genes, given that GnRHa treated boys were reported to show increased testosterone and LH levels.

The absence of an apparent GnRHa stimulation for 11 out of 16 genes remains unexplained, but one possibility could be that they are epigenetically downregulated and therefore unresponsive to GnRHa treatment at the mRNA level. Nonetheless, the absence of GnRHa-responsive key players strengthens and supports the need for alternative pathways, for which we suggest the stimulation of the transcription factors, *EGR2*, *DMRTC2*, *PAX7* and *BRACHYURY*/*T*, the growth factor like proteins, *NRG1* and *NRG3,* and the RNA binding and Y chromosome encoded genes, *RBMY1B*, *RBMY1E*, *RBMY1J* and *TSPY4*.

A clear pattern was not observed regarding the function of GnRHa-responsive and unresponsive genes in germ cell differentiation or gene regulation. While some of the negatively regulated genes are involved in self-renewal (*ETV5* and *ID4*), others control the differentiation process (*DMRT1* and *UCHL1*). Similarly, genes that respond positively to GnRHa treatment are also involved in self-renewal (*PAX7* and *BRACHYURY*/*T*) and differentiation (*EGR2*, *NRG1* and *NRG3*). The differentially expressed genes *EGR2*, *ETV5*, *ID4, TSPAN8* [66,67] and *T* [30] are all regulated by FGF/GDNF signaling, while *FOXO1* [68], *KIT* [69], *NANOS2* [41], *NRG1* and *NRG3* [57], and *PAX7* [70] expressions are regulated by RA. Activated genes of the alternative pathway (*PAX7*, *BRACHYURY*/*T*, *EGR2*, *NRG1*, and *NRG3*) are therefore linked to both FGF/GDNF and RA signaling.

The balance between self-renewal and differentiation depends not only on the described intrinsic factors, but also on extrinsic factors, including GDNF, RA, WNT and testosterone signaling. Spermatogonial cell development in mice depends on testosterone-dependent secretion of GDNF by peritubular myoid cells [71]. *GDNF* expression was significantly increased after GnRHa treatment (logFC^GnRHa^ + 1.47), suggesting expression induced by GnRHa. LH-dependent testosterone secretion was reported to regulate SSC self-renewal by suppressing *Wnt5a* expression in mouse Sertolli cells [72]. GnRHa treated testes showed reduced *WNT5A* expression (logFC^GnRHa^ − 0.86), suggesting a similar testosterone-dependent regulation of SSC self-renewal, by *Wnt5a* suppression in humans.

## 5. Conclusions

Our differential gene expression profiling of gonocyte and spermatogonial markers, particularly *DMRTC2*, *PAX7*, *BRACHYURY*/*T*, and *TERT*, highlights their importance for the development of Ad spermatogonia with specific functionalities in self-renewal and differentiation, and following GnRHa curative treatment. We suggest that GnRHa induced testosterone and a LH increase reconstitute self-renewal properties of the Ad spermatogonial stem cells, and induce RA-responsive genes, such as *NRG1*, *NRG3* and *PAX7*, to help prepare them for commitment to differentiation.

Together with our earlier observations on the level of the HPG-axis of differentially expressed genes in Ad– testes [22,23], we suggest that *EGR4* and *PITX1* controlled by *PROK2/CHD7/FGFR1/SPRY4* genes expression is responsible for LH deficiency, which in turn affects the germ cell transitional effectors, *FGFR3*, *FGF9*, *NANOS2*, *NANOS3*, *SOHLH1* and *SOHLH2*. Upon GnRHa treatment, however, alternative pathways are activated, including the LH-secretion orchestrating factors, *EGR2*, *EGR3*, *TAC1*, *TAC3*, *PROP1* and *LEP,* and the gonocyte-to-Ad spermatogonia transition effectors, *DMRTC2*, *T*, *PAX7*, *TERT*, *NRG1*, *NRG3*, *RBMY1B*, *RBMY1E* and *RBMY1J.*

## Figures and Tables

**Table 1:** Differential expression of gonocyte and spermatogonial marker genes involved in self-renewal and differentiation of spermatogonial stem cells (SSCs). Absolute fold changes (logFC) and false discovery rates (FDR) of differentially expressed genes in the Ad− vs. Ad+ group, and in the GnRHa (Buserelin, INN) treated vs. untreated group are indicated. Absolute fold changes <2 are highlighted in red; n.d.: not determined, n.s.: not significant.

Gene ID	Name	Cell Marker	logFC Ad−/Ad+	FDR Ad−/Ad+	logFC GnRHa	FDR GnRHa
*ALPP/PLAP*	Alkaline phosphatase, placental	gonocytes	n.d.	n.d.	n.s.	n.s.
*EPS8*	Epidermal growth factor receptor pathway substrate 8	gonocytes	n.s.	n.s.	−0.6181	0.0091
*KIT/c-KIT*	KIT proto-oncogene receptor tyrosine kinase	gonocytes	n.s.	n.s.	−0.6307	0.0086
*NANOG*	Nanog homeobox	gonocytes	n.s.	n.s.	n.s.	n.s.
*POU5F1/OCT4*	POU class 5 homeobox 1	gonocytes	−3.0537	0.0059	n.s.	n.s.
*TFAP2C/AP2γ*	Transcription factor AP-2 gamma/activating enhancer binding protein 2 gamma	gonocytes	−2.3598	0.0041	n.s.	n.s.
*ADGRA3/GPR125*	Adhesion G protein-coupled receptor A3/G-protein coupled receptor 125	undifferentiated spermatogonia	n.d.	n.d.	−0.6740	0.0047
*BCL6B*	B-cell CLL/lymphoma 6B	undifferentiated spermatogonia	n.s.	n.s.	n.s.	n.s.
*CDH1*	Cadherin 1	undifferentiated spermatogonia	n.s.	n.s.	n.s.	n.s.
*CHD1L*	Chromodomain helicase DNA binding protein 1-like	undifferentiated spermatogonia	n.s.	n.s.	−0.6220	0.0068
*DAZ1*	Deleted in azoospermia 1	undifferentiated spermatogonia	−2.0896	0.0038	n.s.	n.s.
*DAZL*	Deleted in azoospermia-like	undifferentiated spermatogonia	−1.3031	0.0073	n.s.	n.s.
*DDX4/VASA*	DEAD (Asp-Glu-Ala-Asp) box polypeptide 4	undifferentiated spermatogonia	−2.8616	0.0002	n.s.	n.s.
*DMRT1*	Doublesex and mab-3 related transcription factor 1	undifferentiated spermatogonia	n.s.	n.s.	−0.7838	0.0010
*ETV5*	ETS variant gene 5	undifferentiated spermatogonia	−1.0118	0.0037	−0.4611	0.0490
*FGF9*	Fibroblast growth factor 9	undifferentiated spermatogonia	−1.0605	0.0016	n.s.	n.s.
*FGFR3*	Fibroblast growth factor receptor 3	undifferentiated spermatogonia	−3.3279	0.0002	n.s.	n.s.
*FOXO1*	Forkhead box O1	undifferentiated spermatogonia	n.s.	n.s.	−0.6078	0.0097
*GFRA1*	GDNF family receptor alpha 1	undifferentiated spermatogonia	n.s.	n.s.	n.s.	n.s.
*ID4*	Inhibitor of DNA binding 4	undifferentiated spermatogonia	−1.5342	0.0011	−0.5512	0.0322
*MAGEA4*	Melanoma antigen family A4	undifferentiated spermatogonia	−2.6591	0.0002	n.s.	n.s.
*NANOS2*	Nanos C2HC-type zinc finger 2	undifferentiated spermatogonia	−4.0281	0.0003	n.s.	n.s.
*NANOS3*	Nanos C2HC-type zinc finger 3	undifferentiated spermatogonia	−2.6621	0.0043	n.d.	n.d.
*NEUROG3*	Neurogenin 3	undifferentiated spermatogonia	n.d.	n.d.	n.d.	n.d.
*PAX7*	Paired box 7	undifferentiated spermatogonia	−1.2949	0.0318	1.8592	0.0005
*PHF13/SPOC1*	PHD finger protein 13	undifferentiated spermatogonia	n.s.	n.s.	n.s.	n.s.
*POU2F2/OCT2*	POU class 2 homeobox 2	undifferentiated spermatogonia	n.s.	n.s.	0.9912	0.0166
*RET*	Ret proto-oncogene	undifferentiated spermatogonia	−2.1556	0.0002	n.s.	n.s.
*SALL4*	Spalt-like transcription factor 4	undifferentiated spermatogonia	−1.2253	0.0087	n.s.	n.s.
*SOHLH1*	Spermatogenesis and oogenesis specific basic helix-loop-helix 1	undifferentiated spermatogonia	−2.9639	0.0002	n.s.	n.s.
*SOHLH2*	Spermatogenesis and oogenesis specific basic helix-loop-helix 2	undifferentiated spermatogonia	−1.3457	0.0105	n.s.	n.s.
*T*	T brachyury transcription factor	undifferentiated spermatogonia	−2.4149	0.0146	1.9341	0.0221
*TAF4B*	TATA box binding protein (TBP)-associated factor 4B	undifferentiated spermatogonia	n.s.	n.s.	−0.8142	0.0008
*TERT*	Telomerase reverse transcriptase	undifferentiated spermatogonia	−2.2152	0.0006	1.5623	0.0155
*THY1*	Thy-1 cell surface antigen	undifferentiated spermatogonia	n.s.	n.s.	−0.9577	0.0011
*TSPAN8*	Tetraspanin 8	undifferentiated spermatogonia	n.s.	n.s.	1.1760	0.0154
*TSPY1*	Testis specific protein, Y-linked 1	undifferentiated spermatogonia	−2.4939	0.0003	n.s.	n.s.
*UCHL1*	Ubiquitin C-terminal hydrolase L1	undifferentiated spermatogonia	−1.1036	0.0064	−1.0168	0.0003
*UTF1*	Undifferentiated embryonic cell transcription factor 1	undifferentiated spermatogonia	n.d.	n.d.	n.d.	n.d.
*ZBTB16/PLZF*	Zinc finger and BTB domain containing 16	undifferentiated spermatogonia	n.s.	n.s.	n.s.	n.s.
*DMRTC2/DMRT7*	DMRT-like family C2	spermatogonia?	−1.6666	0.0004	1.0740	0.0199
*EGR2*	Early growth response 2	spermatogonia?	−1.1786	0.0013	1.2310	0.0022
*NRG1*	Neuregulin 1	spermatogonia?	−0.9213	0.0136	0.7797	0.0099
*NRG3*	Neuregulin 3	spermatogonia?	−0.8806	0.0160	0.7177	0.0291
*RBMY1B*	RNA binding motif protein, Y-linked, family 1, member B	spermatogonia?	−1.9326	0.0004	1.1699	0.0023
*RBMY1E*	RNA binding motif protein, Y-linked, family 1, member E	spermatogonia?	−1.9032	0.0020	1.3151	0.0010
*RBMY1J*	RNA binding motif protein, Y-linked, family 1, member J	spermatogonia?	−1.9522	0.0007	0.8343	0.0158
*TSPY4*	Testis specific protein, Y-linked 4	spermatogonia?	−1.9952	0.0004	1.0862	0.0325

## Supplementary Materials

The following are available online at www.mdpi.com/2073-4425/8/10/267/s1, Table S1: Differentially expressed genes involved in the self-renewal of spermatogonial stem cells in HIR/LIR and after GnRHa treatment, Table S2: Differentially expressed genes involved in the differentiation of spermatogonial stem cells in HIR/LIR and after GnRHa treatment.

## References

[1] Forest M.G., Sizonenko P.C., Cathiard A.M., Bertrand J. (1974). Hypophyso-gonadal function in humans during the first year of life. Evidence for testicular activity in early infancy. J. Clin. Investig..

[2] Winter J.S.D., Hughes I.A., Reyes F.I., Faiman C. (1976). Pituitary gonadal relations in infancy: II. Patterns of serum gonadal steroid concentrations in man from birth to two years of age. J. Clin. Endocrinol. Metab..

[3] Corbier P., Edwards D.A., Roffi J. (1992). The neonatal testosterone surge: A comparative study. Arch. Int. Physiol. Biochim. Biophys..

[4] Hadziselimovic F., Thommen L., Girard J., Herzog B. (1986). The significance of postnatal gonadotropin surge for testicular development in normal and cryptorchid testes. J. Urol..

[5] Hadziselimovic F., Emmons L.R., Buser M.W. (2004). A diminished postnatal surge of Ad spermatogonia in cryptorchid infants is additional evidence for hypogonadotropic hypogonadism. Swiss Med. Wkly..

[6] Hadziselimovic F., Zivkovic D., Bica D.T.G., Emmons L.R. (2005). The importance of mini-puberty for fertility in cryptorchidism. J. Urol..

[7] Hadziselimovic F., Herzog B. (2001). The importance of both an early orchidopexy and germ cell maturation for fertility. Lancet.

[8] Hadziselimovic F., Höcht B., Herzog B., Buser M.W. (2007). Infertility in cryptorchidism is linked to the stage of germ cell development at orchidopexy. Horm. Res..

[9] Hadziselimovic F., Höcht B. (2008). Testicular histology related to fertility outcome and postpubertal hormone status in cryptorchidism. Klin. Padiatr..

[10] Hadziselimovic F., Herzog B., Barthold J.S. (1997). Treatment with a luteinizing hormone-releasing hormone analogue after successful orchiopexy markedly improves the chance of fertility later in life. J. Urol..

[11] Hadziselimovic F., Höcht B., Herzog B., Girard J., Labrie F., Belanger A., Dupont A. (1984). Does Long Term Treatment with Buserelin Improve the Fertility Chances of Cryptorchid Testes?. LH-RH and Its Analogues.

[12] Hadziselimovic F., Huff D., Duckett J., Herzog B., Elder J., Snyder H., Buser M. (1987). Treatment of cryptorchidism with low doses of buserelin over a 6-months period. Eur. J. Pediatr..

[13] Hadziselimovic F. (2008). Successful treatment of unilateral cryptorchid boys risking infertility with LH-RH analogue. Int. Braz. J. Urol..

[14] Dym M., Kokkinaki M., He Z. (2009). Spermatogonial stem cells: Mouse and human comparisons. Birth Defects Res. Part C Embryo Today Rev..

[15] Hermann B.P., Sukhwani M., Hansel M.C., Orwig K.E. (2010). Spermatogonial stem cells in higher primates: Are there differences from those in rodents?. Reproduction.

[16] Kersemaekers A.M.F., Honecker F., Stoop H., Cools M., Molier M., Wolffenbuttel K., Bokemeyer C., Li Y., Lau Y.F.C., Oosterhuis J.W. (2005). Identification of germ cells at risk for neoplastic transformation in gonadoblastoma: An immunohistochemical study for OCT3/4 and TSPY. Hum. Pathol..

[17] Rajpert-De Meyts E. (2006). Developmental model for the pathogenesis of testicular carcinoma in situ: Genetic and environmental aspects. Hum. Reprod. Update.

[18] Pesce M., Wang X., Wolgemuth D.J., Scholer H. (1998). Differential expression of the Oct-4 transcription factor during mouse germ cell differentiation. Mech. Dev..

[19] Robinson L.L., Gaskell T.L., Saunders P.T., Anderson R.A. (2001). Germ cell specific expression of c-kit in the human fetal gonad. Mol. Hum. Reprod..

[20] Pauls K., Schorle H., Jeske W., Brehm R., Steger K., Wernert N., Buttner R., Zhou H. (2006). Spatial expression of germ cell markers during maturation of human fetal male gonads: An immunohistochemical study. Hum. Reprod.

[21] Gkountela S., Li Z., Vincent J.J., Zhang K.X., Chen A., Pellegrini M., Clark A.T. (2012). The ontogeny of cKIT+ human primordial germ cells proves to be a resource for human germ line reprogramming, imprint erasure and in vitro differentiation. Nat. Cell Biol..

[22] Hadziselimovic F., Gegenschatz-Schmid K., Verkauskas G., Docampo-Garcia M.J., Demougin P., Bilius V., Malcius D., Dasevicius D., Stadtler M.B. (2016). Gene expression changes underlying idiopathic central hypogonadism in cryptorchidism with defective mini-puberty. Sex. Dev..

[23] Hadziselimovic F., Gegenschatz-Schmid K., Verkauskas G., Demougin P., Bilius V., Dasevicius D., Stadler M.B. (2017). GnRHa Treatment of Cryptorchid Boys Affects Genes Involved in Hormonal Control of the HPG Axis and Fertility. Sex. Dev..

[24] Seguchi H., Hadziselimovic F. (1974). Ultramicroscopic studies on the seminiferous tubule in children from birth to puberty. I. Spermatogonia development. Verh. Anat. Ges..

[25] Szklarczyk D., Franceschini A., Wyder S., Forslund K., Heller D., Huerta-Cepas J., Simonovic M., Roth A., Santos A., Tsafou K.P. (2015). STRING v10: Protein-protein interaction networks, integrated over the tree of life. Nucleic Acids Res..

[26] Hadziselimovic F., Herzog B., Daum R., Mildenberger H., Rehbein F. (1990). Hodenerkrankungen im Kindesalter.

[27] Hadziselimovic F., Hadziselimovic N.O., Demougin P., Krey G., Höcht B., Oakeley E.J. (2009). EGR4 is a master gene responsible for fertility in cryptorchidism. Sex. Dev..

[28] Hadziselimovic F., Hadziselimovic N.O., Demougin P., Oakeley E.J. (2011). Testicular gene expression in cryptorchid boys at risk of azoospermia. Sex. Dev..

[29] Marikawa Y., Tamashiro D.A.A., Fujita T.C., Alarcon V.B. (2011). Dual Roles of Oct4 in the Maintenance of Mouse P19 Embryonal Carcinoma Cells: As Negative Regulator of Wnt/β-Catenin Signaling and Competence Provider for Brachyury Induction. Stem Cells Dev..

[30] Wu X., Goodyear S.M., Tobias J.W., Avarbock M.R., Brinster R.L. (2011). Spermatogonial Stem Cell Self-Renewal Requires ETV5-Mediated Downstream Activation of Brachyury in Mice1. Biol. Reprod..

[31] Rajpert-De Meyts E., Hanstein R., Jørgensen N., Græm N., Vogt P.H., Skakkebæk N.E. (2004). Developmental expression of POU5F1 (OCT-3/4) in normal and dysgenetic human gonads. Hum. Reprod..

[32] Pauls K., Jäger R., Weber S., Wardelmann E., Koch A., Büttner R., Schorle H. (2005). Transcription factor AP-2γ, a novel marker of gonocytes and seminomatous germ cell tumors. Int. J. Cancer.

[33] Tyagi G., Carnes K., Morrow C., Kostereva N.V., Ekman G.C., Meling D.D., Hostetler C., Griswold M., Murphy K.M., Hess R.A. (2009). Loss of Etv5 Decreases Proliferation and RET Levels in Neonatal Mouse Testicular Germ Cells and Causes an Abnormal First Wave of Spermatogenesis. Biol. Reprod..

[34] Chen C., Ouyang W., Grigura V., Zhou Q., Carnes K., Lim H., Zhao G.-Q., Arber S., Kurpios N., Murphy T.L. (2005). ERM is required for transcriptional control of the spermatogonial stem cell niche. Nature.

[35] Bernardo A.S., Faial T., Gardner L., Niakan K.K., Ortmann D., Senner C.E., Callery E.M., Trotter M.W., Hemberger M., Smith J.C. (2011). BRACHYURY and CDX2 Mediate BMP-Induced Differentiation of Human and Mouse Pluripotent Stem Cells into Embryonic and Extraembryonic Lineages. Cell Stem Cell.

[36] Aramaki S., Hayashi K., Kurimoto K., Ohta H., Yabuta Y., Iwanari H., Mochizuki Y., Hamakubo T., Kato Y., Shirahige K. (2013). A Mesodermal Factor, T, Specifies Mouse Germ Cell Fate by Directly Activating Germline Determinants. Dev. Cell.

[37] Eildermann K., Aeckerle N., Debowski K., Godmann M., Christiansen H., Heistermann M., Schweyer S., Bergmann M., Kliesch S., Gromoll J. (2012). Developmental expression of the pluripotency factor sal-like protein 4 in the monkey, human and mouse testis: Restriction to premeiotic germ cells. Cells Tissues Organs.

[38] Gassei K., Orwig K.E. (2013). SALL4 Expression in Gonocytes and Spermatogonial Clones of Postnatal Mouse Testes. PLoS ONE.

[39] Lovelace D.L., Gao Z., Mutoji K., Song Y.C., Ruan J., Hermann B.P. (2016). The regulatory repertoire of PLZF and SALL4 in undifferentiated spermatogonia. Development.

[40] Tsuda M., Sasaoka Y., Kiso M., Abe K., Haraguchi S., Kobayashi S., Saga Y. (2003). Conserved role of nanos proteins in germ cell development. Science.

[41] Suzuki A., Saga Y. (2008). Nanos2 suppresses meiosis and promotes male germ cell differentiation. Genes Dev..

[42] Barrios F., Filipponi D., Pellegrini M., Paronetto M.P., Di Siena S., Geremia R., Rossi P., De Felici M., Jannini E.A., Dolci S. (2010). Opposing effects of retinoic acid and FGF9 on Nanos2 expression and meiotic entry of mouse germ cells. J. Cell Sci..

[43] Tsuda M., Kiso M., Saga Y. (2006). Implication of nanos2-3′UTR in the expression and function of nanos2. Mech. Dev..

[44] Kusz K.M., Tomczyk L., Sajek M., Spik A., Latos-Bielenska A., Jedrzejczak P., Pawelczyk L., Jaruzelska J. (2009). The highly conserved NANOS2 protein: Testis-specific expression and significance for the human male reproduction. Mol. Hum. Reprod..

[45] Zhang T., Murphy M.W., Gearhart M.D., Bardwell V.J., Zarkower D. (2014). The mammalian Doublesex homolog DMRT6 coordinates the transition between mitotic and meiotic developmental programs during spermatogenesis. Development.

[46] Desimio M.G., Campolo F., Dolci S., De Felici M., Farini D. (2015). SOHLH1 and SOHLH2 directly down-regulate stimulated by retinoic acid 8 (STRA8) expression. Cell Cycle.

[47] Braga M., Bhasin S., Jasuja R., Pervin S., Singh R. (2012). Testosterone inhibits transforming growth factor-β signaling during myogenic differentiation and proliferation of mouse satellite cells: Potential role of follistatin in mediating testosterone action. Mol. Cell. Endocrinol..

[48] Lim J., Goriely A., Turner G.D., Ewen K.A., Jacobsen G.K., Graem N., Wilkie A.O., Rajpert-De Meyts E. (2011). OCT2, SSX and SAGE1 reveal the phenotypic heterogeneity of spermatocytic seminoma reflecting distinct subpopulations of spermatogonia. J. Pathol..

[49] Pech M.F., Garbuzov A., Hasegawa K., Sukhwani M., Zhang R.J., Benayoun B.A., Brockman S.A., Lin S., Brunet A., Orwig K.E. (2015). High telomerase is a hallmark of undifferentiated spermatogonia and is required for maintenance of male germline stem cells. Genes Dev..

[50] Mei F., Zhang B., Tang Z.-W., Hou L. (2005). Expression of a telomerase-associated gene in normal, atrophic, and tumorous testes. Chin. Med. Sci. J..

[51] Schrader M., Müller M., Schulze W., Heicappell R., Krause H., Straub B., Miller K. (2002). Quantification of telomerase activity, porphobilinogen deaminase and human telomerase reverse transcriptase mRNA in testicular tissue—New parameters for a molecular diagnostic classification of spermatogenesis disorders. Int. J. Androl..

[52] Schrader M., Müller M., Schulze W., Heicappell R., Krause H., Straub B., Miller K. (2002). Quantification of the expression level of the gene encoding the catalytic subunit of telomerase in testicular tissue specimens predicts successful sperm recovery. Hum. Reprod..

[53] Calado R.T., Yewdell W.T., Wilkerson K.L., Regal J.A., Kajigaya S., Stratakis C.A., Young N.S. (2009). Sex hormones, acting on the TERT gene, increase telomerase activity in human primary hematopoietic cells. Blood.

[54] Pinto F., Pértega-Gomes N., Vizcaíno J.R., Andrade R.P., Cárcano F.M., Reis R.M. (2016). Brachyury as a potential modulator of androgen receptor activity and a key player in therapy resistance in prostate cancer. Oncotarget.

[55] Hamra K.F., Schultz N., Chapman K.M., Grellhesl D.M., Cronkhite J.T., Hammer R.E., Garbers D.L. (2004). Defining the spermatogonial stem cell. Dev. Biol..

[56] Rossi P., Lolicato F., Grimaldi P., Dolci S., Di Sauro A., Filipponi D., Geremia R. (2008). Transcriptome analysis of differentiating spermatogonia stimulated with kit ligand. Gene Expr. Patterns.

[57] Zhang J., Eto K., Honmyou A., Nakao K., Kiyonari H., Abé S. (2011). Neuregulins are essential for spermatogonial proliferation and meiotic initiation in neonatal mouse testis. Development.

[58] Chapman K., Medrano G., Chaudhary J., Hamra F. (2015). NRG1 and KITL signal downstream of retinoic acid in the germline to support soma-free syncytial growth of differentiating spermatogonia. Cell Death Discov..

[59] Saunders P.T.K., Turner J.M.A., Ruggiu M., Taggart M., Burgoyne P.S., Elliott D., Cooke H.J. (2003). Absence of mDazl produces a final block on germ cell development at meiosis. Reproduction.

[60] Lee J., Hong J., Kim E., Kim K., Kim S.W., Krishnamurthy H., Chung S.S.W., Wolgemuth D.J., Rhee K. (2004). Developmental stage-specific expression of Rbm suggests its involvement in early phases of spermatogenesis. Mol. Hum. Reprod..

[61] Elliott D.J., Millar M.R., Oghene K., Ross A., Kiesewetter F., Pryor J., McIntyre M., Hargreave T.B., Saunders P.T., Vogt P.H. (1997). Expression of RBM in the nuclei of human germ cells is dependent on a critical region of the Y chromosome long arm. Proc. Natl. Acad. Sci. USA.

[62] Elliott D.J., Oghene K., Makarov G., Makarova O., Hargreave T.B., Chandley A.C., Eperon I.C., Cooke H.J. (1998). Dynamic changes in the subnuclear organisation of pre-mRNA splicing proteins and RBM during human germ cell development. J. Cell Sci..

[63] Kawamata M., Nishimori K. (2006). Mice deficient in Dmrt7 show infertility with spermatogenic arrest at pachytene stage. FEBS Lett..

[64] Kim S., Namekawa S.H., Niswander L.M., Ward J.O., Lee J.T., Bardwell V.J., Zarkower D. (2007). A Mammal-Specific Doublesex Homolog Associates with Male Sex Chromatin and Is Required for Male Meiosis. PLoS Genet..

[65] Li Y., Zhang D.J., Qiu Y., Kido T., Lau Y.-F.C. (2017). The Y-located proto-oncogene TSPY exacerbates and its X-homologue TSPX inhibits transactivation functions of androgen receptor and its constitutively active variants. Hum. Mol. Genet..

[66] Oatley J.M., Avarbock M.R., Telaranta A.I., Fearon D.T., Brinster R.L. (2006). Identifying genes important for spermatogonial stem cell self-renewal and survival. Proc. Natl. Acad. Sci. USA.

[67] Oatley M.J., Kaucher A.V., Racicot K.E., Oatley J.M. (2011). Inhibitor of DNA Binding 4 Is Expressed Selectively by Single Spermatogonia in the Male Germline and Regulates the Self-Renewal of Spermatogonial Stem Cells in Mice1. Biol. Reprod..

[68] Yu F., Jin L., Yang G., Ji L., Wang F., Lu Z. (2014). Post-transcriptional repression of FOXO1 by QKI results in low levels of FOXO1 expression in breast cancer cells. Oncol. Rep..

[69] Koli S., Mukherjee A., Reddy K.V.R. (2017). Retinoic acid triggers c-kit gene expression in spermatogonial stem cells through an enhanceosome constituted between transcription factor binding sites for retinoic acid response element (RARE), spleen focus forming virus proviral integration oncogene (SPFI1) (PU.1) and E26 transformation-specific (ETS). Reprod. Fertil. Dev..

[70] Colleoni S., Galli C., Gaspar J.A., Meganathan K., Jagtap S., Hescheler J., Sachinidis A., Lazzari G. (2011). Development of a Neural Teratogenicity Test Based on Human Embryonic Stem Cells: Response to Retinoic Acid Exposure. Toxicol. Sci..

[71] Chen L.-Y., Willis W.D., Eddy E.M. (2016). Targeting the Gdnf Gene in peritubular myoid cells disrupts undifferentiated spermatogonial cell development. Proc. Natl. Acad. Sci. USA.

[72] Tanaka T., Kanatsu-Shinohara M., Lei Z., Rao C.V., Shinohara T. (2016). The Luteinizing Hormone-Testosterone Pathway Regulates Mouse Spermatogonial Stem Cell Self-Renewal by Suppressing WNT5A Expression in Sertoli Cells. Stem Cell Rep..

