# Supplementary material for: DMRTC2, PAX7, BRACHYURY/T and TERT Are Implicated in Male Germ Cell Development Following Curative Hormone Treatment for Cryptorchidism-Induced Infertility
Source: Genes (Basel). 2017 Oct 11;8(10):267. doi: 10.3390/genes8100267 (PMC5664117; doi:10.3390/genes8100267)
Supplement: Supplementary file 1 [file genes-08-00267-s001.docx]

| **Gene ID** | **Name** | **logFC Ad-/Ad+** | **FDR Ad-/Ad+** | **logFC GnRHa** | **FDR GnRHa** |
| --- | --- | --- | --- | --- | --- |
| *BCL6B* | B-cell CLL/lymphoma 6B | n.s. | n.s. | n.s. | n.s. |
| *CCNE2* | Cyclin E2 | -1.5570 | 0.0005 | n.s. | n.s. |
| *CDH1* | Cadherin 1 | n.s. | n.s. | n.s. | n.s. |
| *CXCR4* | Chemokine (C-X-C motif) Receptor 4 | -0.8001 | 0.0106 | -0.5964 | 0.0291 |
| *CXCL12* | Chemokine (C-X-C motif) Ligand 12 | n.s. | n.s. | -0.8202 | 0.0038 |
| *ETV5* | ETS Variant 5 | -1.0118 | 0.0037 | -0.4611 | 0.0490 |
| *FBXW7* | F-Box and WD repeat domain containing 7 | -0.6717 | 0.0051 | -0.6651 | 0.0049 |
| *GFRA2* | GDNF Family Receptor Alpha 2 | -0.9372 | 0.0193 | n.s. | n.s. |
| *GFRA3* | GDNF Family Receptor Alpha 3 | -1.3695 | 0.0224 | n.s. | n.s. |
| *ID1* | Inhibitor of DNA binding 1 | -2.1026 | 0.0003 | n.s. | n.s. |
| *ID4* | Inhibitor of DNA binding 4 | -1.5342 | 0.0011 | -0.5512 | 0.0322 |
| *INSL6* | Insulin-Like 6 | -2.0675 | 0.0068 | n.d. | n.d. |
| *LIMK1* | LIM domain Kinase 1 | -1.2922 | 0.0005 | n.s. | n.s. |
| *NRG1* | Neuregulin 1 | -0.9213 | 0.0136 | 0.7797 | 0.0099 |
| *NXF2* | Nuclear RNA export Factor 2 | -2.0595 | 0.0002 | n.s. | n.s. |
| *PAX7* | Paired box 7 | -1.2949 | 0.0318 | 1.8592 | 0.0005 |
| *POU3F1* | POU class 3 homeobox 1 | -2.5351 | 0.0004 | n.s. | n.s. |
| *RET* | RET proto-oncogene | -2.1556 | 0.0002 | n.s. | n.s. |
| *SALL4* | Spalt-like transcription factor 4 | -1.2253 | 0.0087 | n.s. | n.s. |
| *T* | T brachyury transcription factor | -2.4149 | 0.0146 | 1.9341 | 0.0221 |
| *TAF4B* | TATA box binding protein (TBP)-Associated Factor 4B | n.s. | n.s. | -0.8142 | 0.0008 |
| *TERT* | Telomerase Reverse Transcriptase | -2.2152 | 0.0006 | 1.5623 | 0.0155 |
| *UTF1* | Undifferentiated embryonic cell Transcription Factor 1 | n.d. | n.d. | n.d. | n.d. |

**Table S1.** Differentially expressed genes involved in self-renewal of spermatogonial stem cells in Ad-/Ad+ and after GnRHa treatment. Absolute fold change (logFC) and false discovery rate (FDR) of differentially expressed genes in the Ad- vs. Ad+ group (Ad-/Ad+) and in the GnRHa treated vs untreated group (GnRHa) are indicated. Absolute fold changes <2 are highlighted in red. n.d.: not determined, n.s.: not significant.

**Table S2.** Differentially expressed genes involved in the differentiation of spermatogonial stem cells in Ad-/Ad+ and after GnRHa treatment. Absolute fold change (logFC) and false discovery rate (FDR) of differentially expressed genes in the Ad- vs. Ad+ group (Ad-/Ad+) and in the GnRHa treated vs. untreated group (GnRHa) are indicated. Absolute fold changes <2 are highlighted in red. n.d: not determined, n.s.: not significant.

| **Gene ID** | **Name** | **logFC Ad-/Ad+** | **FDR Ad-/Ad+** | **logFC GnRHa** | **FDR GnRHa** |
| --- | --- | --- | --- | --- | --- |
| *ALDH1A2* | Aldehyde Dehydrogenase 1 family, member A2 | -2.3695 | 0.0002 | n.s. | n.s. |
| *CYP26B1* | cytochrome P450, family 26, subfamily B, polypeptide 1 | -1.4423 | 0.0004 | -0.7667 | 0.0024 |
| *DAZ1* | deleted in azoospermia 1 | -2.0896 | 0.0038 | n.s. | n.s. |
| *DAZL* | deleted in azoospermia-like | -1.3031 | 0.0073 | n.s. | n.s. |
| *DMRT1* | doublesex and mab-3 related transcription factor 1 | n.s. | n.s. | -0.7838 | 0.0010 |
| *DMRTB1/DMRT6* | DMRT Like Family B With Proline Rich C-Terminal 1 | -3.2649 | 0.0002 | n.s. | n.s. |
| *DMRTC2/DMRT7* | DMRT-like Family C2 | -1.6666 | 0.0004 | 1.0740 | 0.0199 |
| *EGR2* | Early Growth Response 2 | -1.1786 | 0.0013 | 1.2310 | 0.0022 |
| *EGR3* | Early Growth Response 3 | n.s. | n.s. | 1.5959 | 0.0003 |
| *EGR4* | Early Growth Response 4 | -3.3522 | 0.0003 | n.s. | n.s. |
| *EPCAM* | Epithelial Cell Adhesion Molecule | n.s. | n.s. | -0.8727 | 0.0007 |
| *FGF9* | Fibroblast Growth Factor 9 | -1.0605 | 0.0016 | n.s. | n.s. |
| *FGFR1* | Fibroblast Growth Factor Receptor 1 | -0.4937 | 0.0243 | n.s. | n.s. |
| *FGFR2* | Fibroblast Growth Factor Receptor 2 | -1.2529 | 0.0005 | -0.5732 | 0.0139 |
| *FGFR3* | Fibroblast Growth Factor Receptor 3 | -3.3279 | 0.0002 | n.s. | n.s. |
| *FOXO1* | Forkhead box O1 | n.s. | n.s. | -0.6078 | 0.0097 |
| *KHDRBS3* | KH Domain containing, RNA binding, Signal transduction associated 3 | -0.9079 | 0.0253 | -0.6347 | 0.0104 |
| *LY6K* | Lymphocyte Antigen 6 Family Member K | -2.5334 | 0.0003 | n.s. | n.s. |
| *NANOS2* | Nanos homolog 2 | -4.0281 | 0.0003 | n.s. | n.s. |
| *NANOS3* | Nanos homolog 3 | -2.6621 | 0.0043 | n.d. | n.d. |
| *NEUROG3* | Neurogenin 3 | n.d. | n.d. | n.d. | n.d. |
| *NRG1* | Neuregulin 1 | -0.9213 | 0.0136 | 0.7797 | 0.0099 |
| *NRG3* | Neuregulin 3 | -0.8806 | 0.0160 | 0.7177 | 0.0291 |
| *OPRL1* | Opiate Receptor-like 1 | -2.2255 | 0.0013 | n.s. | n.s. |
| *PIWIL1* | Piwi Like RNA-Mediated Gene Silencing 1 | -2.9784 | 0.0002 | n.s. | n.s. |
| *PIWIL2* | Piwi Like RNA-Mediated Gene Silencing 2 | -1.7587 | 0.0009 | n.s. | n.s. |
| *RB1* | Retinoblastoma 1 | 0.2293 | 0.0247 | -0.9153 | 0.0004 |
| *RBMY1B* | RNA Binding Motif protein, Y-linked, family 1, member B | -1.9326 | 0.0004 | 1.1699 | 0.0023 |
| *RBMY1E* | RNA Binding Motif protein, Y-linked, family 1, member E | -1.9032 | 0.0020 | 1.3151 | 0.0010 |
| *RBMY1F* | RNA Binding Motif protein, Y-linked, family 1, member F | -2.0282 | 0.0008 | n.s. | n.s. |
| *RBMY1J* | RNA Binding Motif protein, Y-linked, family 1, member J | -1.9522 | 0.0007 | 0.8343 | 0.0158 |
| *SOHLH1* | Spermatogenesis and Oogenesis specific basic Helix-Loop-Helix 1 | -2.9639 | 0.0002 | n.s. | n.s. |
| *SOHLH2* | Spermatogenesis and Oogenesis specific basic Helix-Loop-Helix 2 | -1.3457 | 0.0105 | n.s. | n.s. |
| *SOX3* | SRY (sex determining region Y)-box 3 | n.d. | n.d. | n.d. | n.d. |
| *SPATA22* | Spermatogenesis Associated 22 | -0.9485 | 0.0033 | n.s. | n.s. |
| *SPO11* | SPO11 meiotic protein covalently bound to DSB | -2.7677 | 0.0002 | n.s. | n.s. |
| *STAT3* | Signal Transducer And Activator Of Transcription 3 | n.s. | n.s. | -0.5348 | 0.0263 |
| *SYCP1* | Synaptonemal Complex Protein 1 | -1.1737 | 0.0111 | n.s. | n.s. |
| *SYCP2* | Synaptonemal Complex Protein 2 | -1.6479 | 0.0013 | n.s. | n.s. |
| *TEX11* | Testis Expressed 11 | -2.2756 | 0.0002 | n.s. | n.s. |
| *TEX13B* | Testis Expressed 13B | -2.8591 | 0.0013 | n.d. | n.d. |
| *TEX14* | Testis Expressed 14 | -1.5375 | 0.0005 | n.s. | n.s. |
| *TEX15* | Testis Expressed 15 | -1.5810 | 0.0005 | -0.8460 | 0.0015 |
| *TEX37* | Testis Expressed 37 | -3.6463 | 0.0003 | n.s. | n.s. |
| *TEX101* | Testis Expressed 101 | -1.7600 | 0.0015 | n.s. | n.s. |
| *THY1* | Thy-1 cell surface antigen | n.s. | n.s. | -0.9577 | 0.0011 |
| *TSPAN8* | Tetraspanin 8 | n.s. | n.s. | 1.1760 | 0.0154 |
| *TSPY1* | Testis Specific Protein, Y-linked 1 | -2.4939 | 0.0003 | n.s. | n.s. |
| *TSPY2* | Testis Specific Protein, Y-linked 2 | -2.6835 | 0.0002 | n.s. | n.s. |
| *TSPY3* | Testis Specific Protein, Y-linked 3 | -2.2866 | 0.0005 | n.s. | n.s. |
| *TSPY4* | Testis Specific Protein, Y-linked 4 | -1.9952 | 0.0004 | 1.0862 | 0.0325 |
| *TSPY8* | Testis Specific Protein, Y-linked 8 | -2.8421 | 0.0002 | n.s. | n.s. |
| *UCHL1* | Ubiquitin C-Terminal Hydrolase L1 | -1.1036 | 0.0072 | -1.0168 | 0.0003 |
| *VRK1* | Vaccinia Related Kinase 1 | -0.8119 | 0.0024 | -0.9047 | 0.0007 |
| *ZBTB16/PLZF* | Zinc finger and BTB domain containing 16 | n.s. | n.s. | n.s. | n.s. |
